# Critical ischaemia of the hand and upper limb in a patient with long COVID-19 infection

**DOI:** 10.1177/17531934211014358

**Published:** 2021-05-13

**Authors:** Vivien C. Lees, Jason K. F. Wong, Ibrahim Ibrahim

**Affiliations:** 1 Department of Plastic and Reconstructive Surgery, Manchester University NHS Foundation Trust, Manchester, UK; 2 Institute of Inflammation and Repair, University of Manchester, Manchester, UK

Dear Editor,

We report the case of a 31-year-old, right-hand dominant female, who presented with critical ischaemia of the dominant hand following iatrogenic injury to the ulna artery at the wrist, during attempted venepuncture to monitor renal function (after a recent kidney infection). Past medical history included a clinical history of COVID-19 infection 5 months previously and occasional chronic pyelonephritis over 8 years. The patient was taking the combined oral contraceptive pill and her body mass index (BMI) was 25.7.

**Figure 1.**
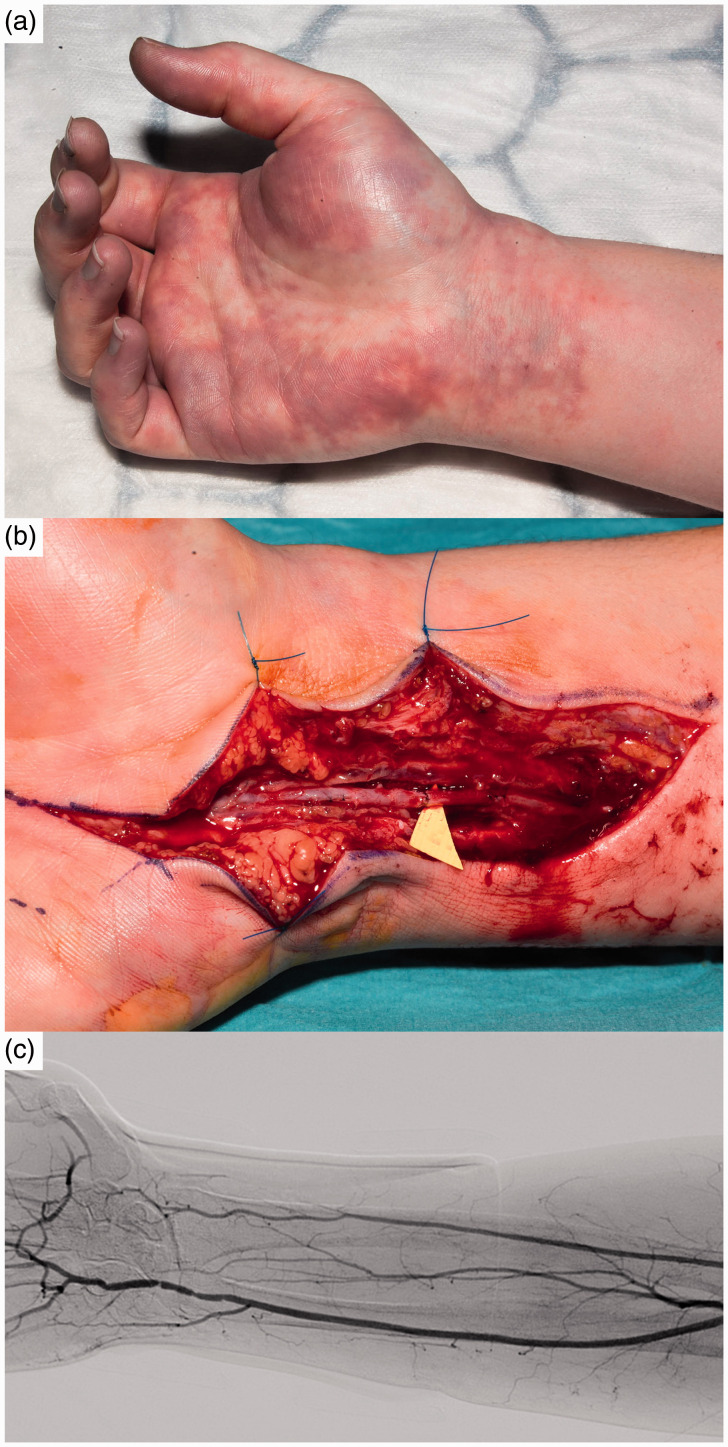
(a) Preoperative image of right hand on presentation. (b) Intra-operative image of right ulna artery primary repair following thrombectomy. (c) Right upper limb angiography demonstrating patency at ulna artery repair and absent distal segment of right radial artery.

The patient then applied a heartrate/motion tracker band to the wrist and the hand subsequently became cool, swollen, mottled and excruciatingly painful. Removal of the fitness tracker band did not improve matters (Figure 1(a)). She presented to the Emergency Department and a duplex ultrasound scan confirmed occlusion of the ulna artery at the wrist, with no flow through the radial artery and no distal run-off to the digits. Almost 24 hours after the initial venepuncture injury, the patient was referred to the plastic surgery service and underwent emergency exploration of the ulna artery. At operation, the artery was found thrombosed at the site of venepuncture. Thrombus was removed, the damaged segment resected and the artery successfully primarily anastomosed (Figure 1(b)). Despite early improvement and continuing patency of the ulna artery, the hand continued to demonstrate poor perfusion, particularly affecting thumb, index and middle fingers distally – interpreted to be related to distal thrombosis.

The next day, an upper limb arteriogram was performed, which revealed absent perfusion of a 4–5 cm distal segment of the radial artery and no distal run-off in the digital vessels (Figure 1(c)). Thrombolysis treatment was commenced with tissue plasminogen activator infusion, through an indwelling brachial artery catheter demonstrating improvement in perfusion status. A repeat angiogram was undertaken at 24 hours for lysis check and while removing the catheter, the mid-section of the brachial artery occluded with further thrombus, followed by the proximal and mid-sections of the ulna artery. After thrombectomy of both brachial and ulna arteries by the interventional radiologist, the indwelling catheter was removed and further thrombolysis discontinued. Immediately following thrombectomy, the hand was clinically ischaemic and appeared white, with now absent pulses and doppler signals. Clinically, further thrombosis had occurred, notwithstanding ongoing therapeutic heparin infusion. Subsequent emergency surgical exploration, extensive removal of adventitia and inspection, revealed axillary artery thrombus extending to the distal bifurcation of the brachial artery, at the radial and ulna arteries. Axillary and brachial artery thrombectomy was performed through a longitudinal arteriotomy using a Fogarty catheter. An ulna artery jump graft using the long saphenous vein was sited from the brachial artery at elbow level to the ulna artery at wrist level, successfully restoring antegrade flow to the palmar arch, demonstrated through positive intra-operative digital doppler signals and finger perfusion, excepting the tips. Two weeks later, on a further visit to theatre, direct visualization revealed the radial artery was maintaining perfusion to the entire hand, as the brachial–ulna bypass graft had thrombosed, despite anticoagulation. Hand and limb perfusion were restored albeit with ultimate loss of the distal tips of the index and middle fingers, which have healed following an interval debridement.

A comprehensive haematological investigation was undertaken for precipitants of hypercoagulability, including Protein C, Lupus anticoagulant, anticardiolipin antibody and antiphospholipid antibody, which were all negative. There was no past or family history of thromboembolic disorders.

The patient was warfarinized and advised to discontinue the oral contraceptive pill. She then became pregnant and re-attended hospital 3 months later with a threatened miscarriage of a first trimester pregnancy, spontaneous deep vein thrombosis of the knee and a clinically diagnosed pulmonary embolus. She remained refractory to therapeutic dose, low molecular weight heparin and aspirin, suggesting the patient remained in a hypercoagulable state. An echocardiogram was unremarkable and platelet counts were normal throughout the duration of the inpatient episodes.

Five months prior to presentation, the patient had contracted COVID-19 infection. Although patients were not being routinely tested at this stage, she developed symptoms of ageusia, fever, cough, shortness of breath and fatigue persisting for a period of 5 weeks and requiring overnight hospital admission for low oxygen saturations. The haematology opinion is that previous COVID-19 infection and the longstanding effects of COVID-19 was likely a significant causative factor in the highly unusual hypercoagulable state, with predisposition to arterial thrombotic sequence that would not have normally arisen from a needle puncture.

The link between acute COVID-19 infection and the hypercoagulable state has been previously described in clinically obtunded patients and correlated with a generally poor prognosis (Levi et al., 2020). Previous reports of critical limb ischaemia in COVID-19 infection have typically been in the high-dependency setting with multi-organ failure and with inotropic support (Qian and Pan, 2020; Schultz and Wolf, 2020; Thiel et al., 2021). Our case is notable, as we describe critical limb ischaemia as the presenting condition 5 months after COVID-19 infection. Arterial thrombosis of the upper limb may be associated with long COVID-19.
